# Oral exposure to thiacloprid-based pesticide (Calypso SC480) causes physical poisoning symptoms and impairs the cognitive abilities of bumble bees

**DOI:** 10.1186/s12862-023-02111-3

**Published:** 2023-04-05

**Authors:** Lotta Kaila, Anna Antinoja, Marjaana Toivonen, Marja Jalli, Olli J. Loukola

**Affiliations:** 1 Department of Agricultural Sciences, University of Helsinki, P.O. Box 27, 00014 Helsinki, Finland; 2 Natural Resources Institute Finland (Luke), Latokartanonkaari 9, 00790 Helsinki, Finland; 3 Ecology and Genetics Research Unit, University of Oulu, PO Box 3000, 90014 Oulu, Finland; 4 Biology Centre of the Czech Academy of Sciences, Inst of Entomology, and Univ. of South Bohemia, Faculty of Science, Ceske Budejovice, Czech Republic; 5 Finnish Environment Institute (SYKE), Biodiversity Centre, Latokartanonkaari 11, 00790 Helsinki, Finland; 6 Natural Resources Institute Finland (Luke), Tietotie 4, 31600 Jokioinen, Finland; 7 Biodiversity Unit, University of Oulu, University of Oulu, PO Box 3000, 90014 Oulu, Finland

**Keywords:** Bumble bee, Associative learning, *Bombus terrestris*, Insecticide exposure, Neonicotinoids, Pollinator, Sublethal effects

## Abstract

**Background:**

Pesticides are identified as one of the major reasons for the global pollinator decline. However, the sublethal effects of pesticide residue levels found in pollen and nectar on pollinators have been studied little. The aim of our research was to study whether oral exposure to the thiacloprid levels found in pollen and nectar affect the learning and long-term memory of bumble bees. We tested the effects of two exposure levels of thiacloprid-based pesticide (Calypso SC480) on buff-tailed bumble bee (*Bombus terrestris*) in laboratory utilizing a learning performance and memory tasks designed to be difficult enough to reveal large variations across the individuals.

**Results:**

The lower exposure level of the thiacloprid-based pesticide impaired the bees’ learning performance but not long-term memory compared to the untreated controls. The higher exposure level caused severe acute symptoms, due to which we were not able to test the learning and memory.

**Conclusions:**

Our results show that oral exposure to a thiacloprid-based pesticide, calculated based on residue levels found in pollen and nectar, not only causes sublethal effects but also acute lethal effects on bumble bees. Our study underlines an urgent demand for better understanding of pesticide residues in the environment, and of the effects of those residue levels on pollinators. These findings fill the gap in the existing knowledge and help the scientific community and policymakers to enhance the sustainable use of pesticides.

## Introduction

Food production security is balancing between favouring beneficial pollinating insects and avoiding intensive invasions of harmful pest insects. In agroecosystems, pollinators offer essential ecosystem services, contributing to the yield of 75% of the leading global food crops [24]. However, the control of insect pests in current plant production relies strongly on insecticides, many of which are not selective but harm all kinds of insects including beneficial ones like pollinators [3]. According to the Food and Agriculture Organization of the United Nations (FAO), the volume of globally used insecticides has been relative stable in the last decade, but insecticide use has been increasing in developing countries as agricultural production has risen in these regions [14].

The intergovernmental science-policy platform on biodiversity and ecosystem services (IPBES) identified insecticides as one of the reasons for pollinator decline [20]. The effects of insecticide use on pollinators depend, among others, on the active substances and co-formulants used, as well as the exposure level of pollinators [13]. Acute and chronic toxicity of insecticides, measured as the exposure level where half of the tested honey bees (*Apis mellifera)* die, have been studied as part of the risk assessment of insecticides at least in the European Union (EU) [13]. However, the sublethal effects of smaller exposure levels on honey bees and other pollinators have been studied substantially less [22].

Neonicotinoids are the most widely used class of insecticides globally [43]. They are also the most studied insecticide group, at least as regards to studies on the effects of pesticides on honey bees [2] and the residue levels in their food, pollen and nectar [44]. The pesticide group includes active substances such as acetamiprid, clothianidin, imidacloprid, thiacloprid and thiamethoxam. Neonicotinoids in sublethal doses have been shown to have negative impacts on bees, including reduced foraging ability, colony growth, reproduction, immunocompetence and foraging motivation [37, 1, 17, 29, 36, 42, 43].

Despite of the known risks of insecticides on pollinators, the knowledge on pesticide residues in pollen, nectar and other bee-related matrices is limited [2, 44]. Since pesticide residues and exposure levels of pollinators in the environment are poorly known, it is hard to fully understand the effects of pesticide use on pollinators [22]. Furthermore, the lack of knowledge on the residue levels leaves space for debate whether the tested pesticide doses are field-realistic or not [6, 7, 35]

Our aim in this study was to examine whether sublethal oral exposure to a thiacloprid-based pesticide affects the learning and long-term memory of bumble bees. The studied exposure levels were based on residues found in pollen [22] and nectar [23] in Finnish agricultural landscape next to oilseed rape cultivations where pesticides were used according to good agricultural practices including respecting the risk mitigation methods set by the national authorities. To study the cognitive abilities, we utilized a 10-colour learning paradigm designed and shown to result in large variations in learning and memory performances across bumble bee individuals [26]. The study increases the knowledge on sublethal effects of pesticide residue levels found in pollen and nectar in order to better understand the consequences of pesticide use for pollinators and pollination services.

## Results

### Symptoms after the lower pesticide exposure

After the pesticide exposure, all the 17 control bees (treatment 0) returned to their hive normally (returned immediately after consuming the sucrose solution by themselves or were removed with a cup to the corridor from where they independently entered the hive). By contrast, most of the 20 bees treated with 0.10 µg thiacloprid per bee (treatment 1) did not return to their hive normally or had other visible symptoms after the exposure. 12 out of 20 bees from treatment 1 stayed in the corridor 30–90 min before entering the hive, six bees stayed on their back for several minutes, four hung their proboscises, one vomited and one walked in a circle in the hall section the next day. Only five out of 20 bees from treatment 1 did not have any visible symptoms and behaved as the control bees did. 17 bees from treatment 1 were trained in the learning phase and tested in the memory test.

### Symptoms after the higher pesticide exposure

**Fig. 1:**
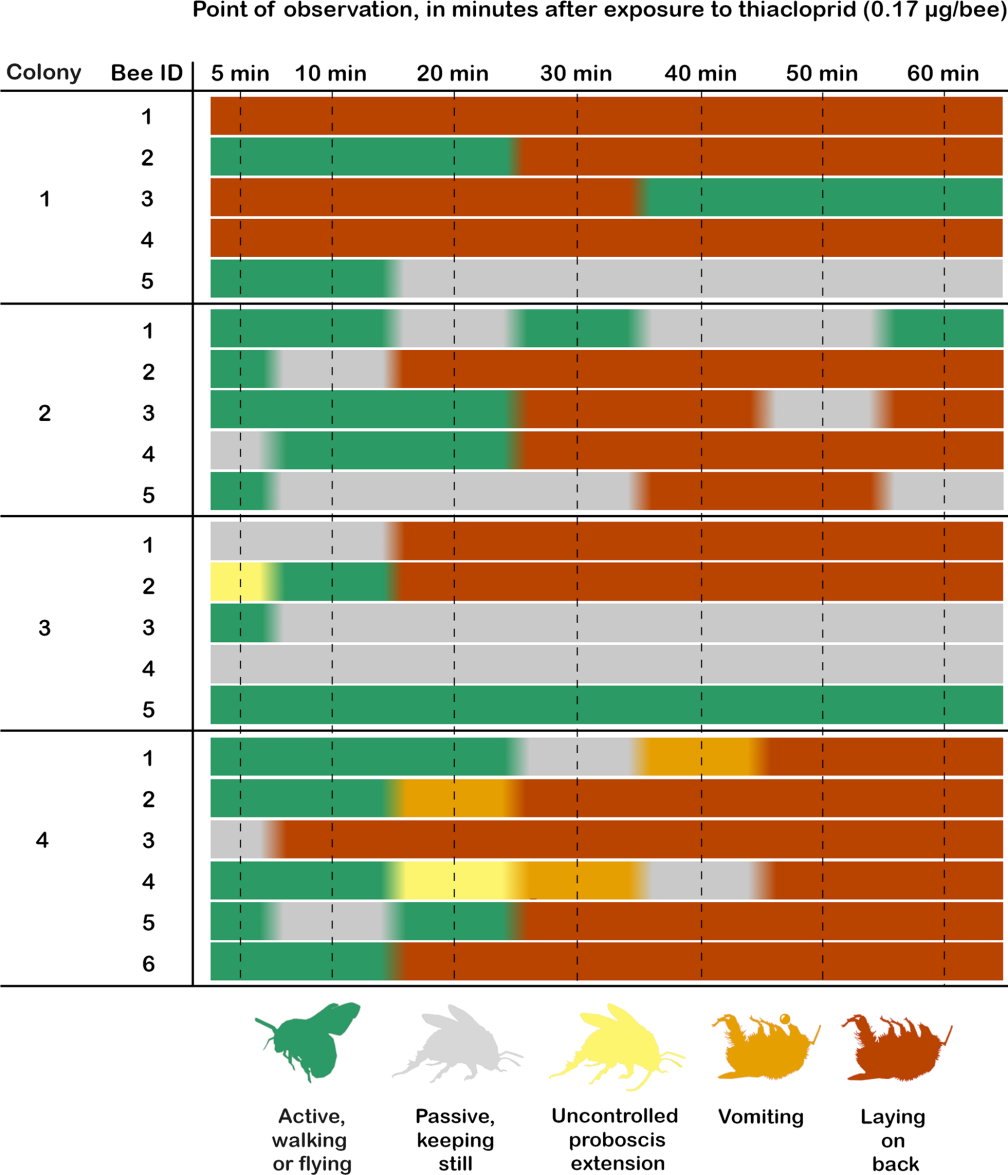
The figure illustrates the symptoms observed in ten minutes intervals for 1 h after the thiacloprid exposure of the studied bumble bees in treatment 2

Of the 21 bees treated with 0.17 µg thiacloprid per bee (treatment 2), 16 bees stayed on their back for at least 20 min of the one-hour’s observation period (Fig. 1). Only one bee was active during the whole observation period, and additionally, three bees did not flip to their back but were passive during most of the observation period. Other observed symptoms varied between the colonies so that only individuals from colony four vomited, and only two bees hung their tongues (one from colony 3 and the other from colony 4). Bees from treatment 2 were not trained in the learning phase or tested in the memory test due to the abovementioned physical symptoms.

### The effects of pesticide exposure on the learning and memory of bees

**Fig. 2:**
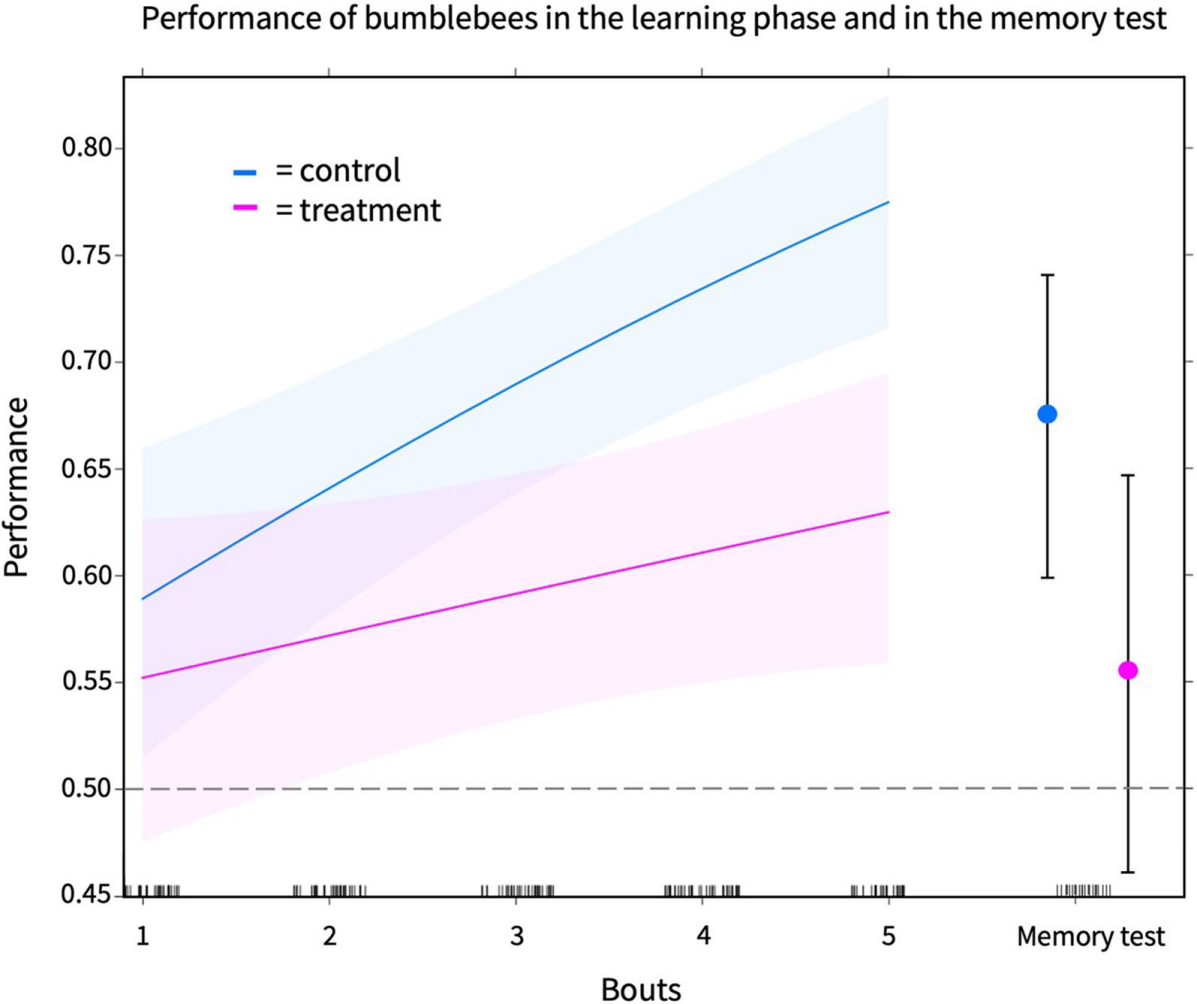
The left side of the figure shows the predicted levels of performance (proportion of correct decisions) and its 95% confidence band for the sample values of performance in the learning phase. Circles on the right side of the figure represent model estimates of performance and error bars represent confidence levels at 95% for the sample values of performance in the memory test. Black bars above each learning bout represents the number of sample values per bout. The horizontal dashed line indicates the chance level (50%)

Model 1. shows that bees’ performance (proportion of correct decisions) increased over learning bouts in general (GLMM; n = 34 (17 controls and 17 treated bees), estimate (bout (learning bouts 1–5)) = 0.218, SE = 0.19, z = 0.73, p =  < 0.01, Fig. 2: left side) but the thiacloprid treatment was negatively associated with the performance (GLMM; estimate (bout (learning bouts 1–5): thiacloprid treatment) = − 0.138, SE = 0.07, z = − 1.98, p =  < 0.05, Fig. 2: left side).

Model 2. shows that there was no significant difference in memory retention from the fifth learning bout to the memory test between the treatments (GLMM; n = 34 (17 controls and 17 treated bees), estimate (bout (learning bout 5 and memory test): thiacloprid treatment) =  − 0.01, SE = 0.31, z = − 0.02, p = 0.98, Fig. 2: right side).

## Discussion

In this study, we show that acute oral exposure to thiacloprid residue levels found in pollen and nectar in agricultural environments has lethal and sublethal effects on bumble bees in laboratory conditions. Our results are particularly remarkable because the thiacloprid residues were found in environments where the use of pesticides followed good agricultural practices. The lethal and sublethal effects reported in our study challenge the protectiveness of the risk mitigation methods as well as underline the urgent need for more knowledge on the pesticide residues in the environment and the accurate methods to study the sublethal effects of pesticides on bumble bees when the products are authorized.

The maximum calculated thiacloprid exposure level (0.17 µg thiacloprid/bee) paralyzed most of the tested bees for minimum of 20 min or caused observable physical poisoning symptoms but some of the bees recovered after the exposure and showed no apparent symptoms. The symptoms are consistent with the mechanism of action of thiacloprid: as all neonicotinoids, thiacloprid is a neurotoxin that binds as agonist to the insect nicotinic acetylcholine receptors (nAChR) causing hyperstimulation and paralysis [39]. Recovering from the symptoms may be possible after the bees have cleaned the substance from their bodies [10].

The lower exposure level (0.10 µg thiacloprid/bee) did not kill the bees but impaired their performance in the learning phase. nAChR plays a central role in mushroom bodies [12], a specific neural region in the bee brain associated with learning and memory [11, 19]. Thus, the reduced learning performance after the thiacloprid exposure may be due to impaired development and function of the mushroom bodies [31, 32]. The exact mechanisms of thiacloprid action on learning and memory processes in the bee brain are unknown. Our results suggest that low thiacloprid exposure disturbs the learning process but may not affect long-term memory of bumble bees. Alternatively, the effects of thiacloprid were temporary, and the bees had recovered from the exposure before the memory test that was conducted two days after the exposure. This would be in line with Cresswell et al. [10], who reported that the activity and feeding of bumble bees returned to normal in 48 h after the exposure to imidacloprid, another active substance in the group of neonicotinoids.

After the lower exposure, the bees did not return to their hive normally as did the untreated control bees. This may be explained by the altruistic self-removal of sick individuals from their colonies, a phenomenon described for social insects [34]. Furthermore, recent research has suggested that thiacloprid causes transcriptional changes in gene associated with mitochondria, which in turn affects metabolism and energy allocation in the bee brain [16]. The alterations in the energy metabolism in the brain can translate to various physiological and behavioural changes [9, 16, 27] and may be one reason for the reduced homing capacity, as well as for the reduced learning performance, after the lower exposure.

Associative learning and memory are essential for the bees to efficiently collect multiple resources from a diversity of flower species [8]. Our findings underline that, when studying bumble bee learning and memory, it is important to use tasks difficult enough resulting in large variations in learning and memory performances across individuals, and thus revealing potential sublethal effects of field-realistic doses. The commonly applied two-colour visual discrimination task [21, 25, 28, 30] may not be difficult enough to reveal variation in learning speed and memory retention between individuals when colours are easily distinguishable.

In nature, the effects of insecticides observed in this study can have serious detrimental consequences for the success of bumble bee colonies and their ability to deliver pollination services. Reduced cognitive skills caused by the thiacloprid exposure are likely to impair bumble bee workers’ foraging efficiency, which negatively affects colony growth and survival [5, 18]. In fact, negative effects of neonicotinoids on foraging efficiency, and changes in foraging preferences of free-flying bumble bees have been reported in several studies [15, 17, 36]. The production of new queens can also suffer as a result of reduced pollen supply to the colony [15, 41]. In addition, temporary paralysis caused by the higher thiacloprid exposure level can increase the mortality of bumble bee workers through predation.

It should be noted that, in our study, we exposed the bees to a commercial pesticide formulation Calypso SC480, not to pure thiacloprid. A recent study by Straw and Brown [38] found that, instead of an active substance, a co-formulant of a pesticide was behind the sublethal effects on bumble bees. Thus, we cannot rule out that the symptoms reported in our study are caused by the co-formulants of Calypso SC480 instead of, or along with, thiacloprid. The EU has banned the use of thiacloprid in open fields after 2020 mainly due to the risks for human health and groundwater (European Food Safety Authority, 2019). However, the active substance is in use outside the EU and besides, the EU member states may grant emergency authorizations for pesticides that are not authorized. The ongoing use of thiacloprid-based pesticides and the recognized failure of the EU pesticide legislation to consider the effects of pesticide co-formulants [38] highlight the need of developing the pesticide legislation, and of studying the sublethal effects of both active substances and co-formulants on pollinators.

Several studies report studying the exposure of bumble bees or honey bees to *field-realistic* pesticide levels. Despite this, there is no standardized way of exposing the bees to pesticides which makes it difficult to compare the studies. Field-realistic exposure levels may be based on residues found in pollen (for example [1, 36, 40] or nectar (for example [1], the application rate sprayed on the field (for example [40], or a combination of these three. We find that the most realistic oral exposure levels are based on the residue levels in nectar (the main food source of bumble bees) and pollen (the secondary food source of bumble bees) [13], and thus calculated the exposure level based on thiacloprid residues found in these matrices [22, 23].

Unlike our study, previous studies on the effects of field-realistic pesticide exposure have used gravity feeders containing sucrose solution and pesticide,and the bumble bees have had free access to the liquid. The benefit of the gravity feeder is that the bumble bees may eat as much as they want, while the weakness is that the exact amount consumed by each individual is not always known. Instead of the gravity feeder, in our study, the bumble bees were exposed individually to thiacloprid based on their calculated daily nectar and pollen consumption and thiacloprid residues found in these matrices. The thiacloprid exposure was given to the bees as a single acute dose to ensure the exact exposure level of each tested individual. In nature, however, the bumble bees might not expose to such high dose levels of thiacloprid as used here because the active substance might hinder their foraging already before they reach the full daily exposure that was used in our study. Thus, the acute toxicity symptoms reported during the one-hour observation period after the exposure may not have occurred in nature, because the sublethal effects of thiacloprid had hindered the foraging.

Our study has a few limitations that should be considered when interpreting the results. One was that the body mass of the bumble bees may have affected the results, but this was controlled for by randomly selecting forager bees. Another limitation was that the mechanisms by which the pesticide affected the bees were not investigated, including the possible effect of pesticide exposure on the antenna. Lastly, we did not measure the proportion of bees that consumed all the rewarding chips in the learning phase. Some bees may have been more efficient than others in visiting the rewarding chips, which may have affected their subsequent performance in the memory retention test. Nonetheless, this study provides valuable insights into the effects of thiacloprid on bumble bees, highlighting the need for further research to understand the impacts of pesticides on pollinators, and to develop effective conservation strategies.

## Conclusions

Our study shows that pesticide residues in pollen and nectar in agricultural landscapes may have lethal and sublethal effects on bumble bees even though the residue levels were found in environments where the use of pesticides followed good agricultural practices. These effects may have serious detrimental consequences for the success of bumble bee colonies and their ability to deliver pollination services. The study helps the scientific community and policymakers to further sustainable agriculture by providing information about the effects of field-realistic pesticide levels on bumble bees. Specific to the EU, the study provides the decision makers with detailed information about the success of pesticide risk assessment which helps them to balance between the pros and cons of the use of pesticides when implicating the broad EU pesticide Regulation. Our results also support including the used 10-colour learning paradigm in the future risk assessment of pesticides. There is a need for more knowledge on pesticide residues in the environment, and on how those residues affect pollinators, as well as for more standardized methods for studying the sublethal effects of field-realistic pesticide exposure on pollinators.

## Materials and methods

### Study species

Five buff-tailed bumble bee (*Bombus terrestris*) colonies were purchased from Koppert (Natupol®, Koppert, The Netherlands). On the same day the colonies arrived in the laboratory, they were transferred in wooden nests (31 × 13.5 × 11.5[height] cm) that were divided into a nest section and a hall section containing litter. Before and after the experiments, bees were fed every day with 40% sucrose solution (w/v) and every second day with approximately 7 g of commercial pollen (Koppert B.V., Berkel en Rodenrijs, The Netherlands).

The nests were connected to wooden flight arenas (60 × 40 × 25 [height] cm) with plastic tubes, “corridors” (25 × 4 × 4.5 [height] cm), containing doors that allowed controlling the entering of each bee to the arena. Identities of the forager bees were tracked with individual number tags (Opalithplättchen, Warnholz & Bienenvoigt, Ellerau, Germany) that were attached to the top of their thorax by Super Glue Gel (Loctite, OH, USA).

### Pesticide exposure

The doses for pesticide exposure were calculated based on maximum thiacloprid residues found in honey bee-collected pollen [22] and in honey bee-collected nectar [23] in field conditions in Finland (Table 1) where thiacloprid was used according to the commercial products label texts. Since the sugar concentration in nectar varies, two different doses were chosen. The lower dose was calculated based on an assumption that the bees consumed nectar that contained 30% sugar, and the higher dose assumed that the bees consumed nectar that contained 15% sugar [13].

**Table 1:** Daily oral exposure of adult bees based on the thiacloprid residues in pollen and nectar

Source of residues	Thiacloprid residues (µg/mg)	Sugar concentration of nectar (%)	Bee's daily maximum consumption (mg)***	Daily oral exposure (µg)
Pollen	0.001484*	–	30.30***	0.04
Nectar	0.00013**	15	993.33***	0.13
Nectar	0.00013**	30	496.66	0.06

The exposure is calculated by combining daily oral exposure from pollen and nectar. The sugar concentration of nectar affects the exposure so that the exposure level is 0.10 µg thiacloprid/bee (0.04 µg + 0.06 µg) when the sugar concentration is expected to be 30% and 0.17 µg thiacloprid/bee (0.04 µg + 0.13 µg) when the sugar concentration is expected to be 15%

^*^[22]

^**^[23]

^***^[13]

Commercial product Calypso SC480 (Finnish registration number 2890) containing thiacloprid 480 g/l was used in the study. The bees were exposed by feeding them 10 µl either of: control 0) only 40% sucrose solution, treatment 1) 40% sucrose solution with 0.10 µg thiacloprid from Calypso SC480, or treatment 2) 40% sucrose solution with 0.17 µg thiacloprid from Calypso SC 480 (Table 2). The 10 µl was pipetted from 100 ml stock solutions consisting of: control 0) 40% sucrose solution, treatment 1) 2.3 µl Calypso SC480 and 40% sucrose solution and treatment 2) 3.6 µl Calypso SC 480 and 40% sucrose solution. The number of bees in the treatments was: control 0) 17 bees (from five colonies), treatment 1) 20 bees (from five colonies), and treatment 2) 21 bees (from four colonies).

**Table 2:** The doses of thiacloprid, Calypso SC480 and sucrose solution for each bee in the treatments

Treatment	Thiacloprid µg/bee	Calypso SC480 µl/bee	40% sucrose solution µl/bee
0	0.00	0.00	10
1	0.10	0.00023	9.99977
2	0.17	0.00036	9.99964

The 10 µl dose was served individually to each bee in the flight arena on a plastic chip, and the bee landed next to the dose freely. The dose was pipetted just before the exposure, to prevent the evaporation of the liquid. After the landing, the bee was kept under a cup for 5 min to ensure that the whole dose was consumed by the bee. All the tested bees consumed the whole 10 µl of sucrose solution without hesitation. After being released from under the cup, the bee was allowed to eat pure 40% sucrose solution as much as it wanted. To ensure that the control bees in each colony were not exposed to thiacloprid in the nest, we first assessed the control treatment group for each colony.

The bees were observed for 1 h after the exposure. In the treatments 0 and 1, the return of the bees to the hive, and the general condition of the bees were observed. In the treatment 2, the bees were observed in a more detailed manner due to severe symptoms (see chapter 2.4).

### Experimental approach

The effects of pesticide exposure on the learning and memory of bumble bees were studied using the bees of the treatments 0 (no thiacloprid) and 1 (0.10 µg thiacloprid). In the treatment 2 (0.17 µg thiacloprid), the planned experiments could not be conducted due to severe acute symptoms of the bees after the pesticide exposure (see chapter 2.4). The learning and memory of bumble bees were studied utilising a 10-colour learning paradigm established by Li et al. [26], where bees are challenged to distinguish five different coloured chips with rewarding sucrose solution from five different coloured chips with aversive quinine solution. The experiment consisted of three phases (1) pre-training, (2) learning phase and (3) memory test. The studied bees were active foragers, and their treatment groups were randomized. All the experiments were conducted in the spring of 2021, between 9 a.m. and 6 p.m. under standardized light (LED, 2700K, 230 VAC) and temperature (25 ± 5 °C) conditions at the Bee laboratory at Botanical Gardens of Oulu University (Finland).

#### Pre-training

The study bees were pre-trained to land on transparent chips (2.5 × 2.5. × 5 [height] cm) with a drop of 7 µl 40% sucrose solution. Ten chips were placed randomly in the flight arena. The pre-training consisted of five foraging bouts per studied bee. After each pre-training bout, the bee entered the hive and emptied its honey crop before the next bout. Between the bouts, the arena and the chips were cleaned with 70% ethanol in water, and the chips were re-filled. Only bees that actively foraged sucrose solution passed the pre-training phase (all the tested bees were active foragers), but the size of the bees was not measured.

The pesticide exposure was done after the pre-training phase.

#### Learning phase

**Fig. 3:**
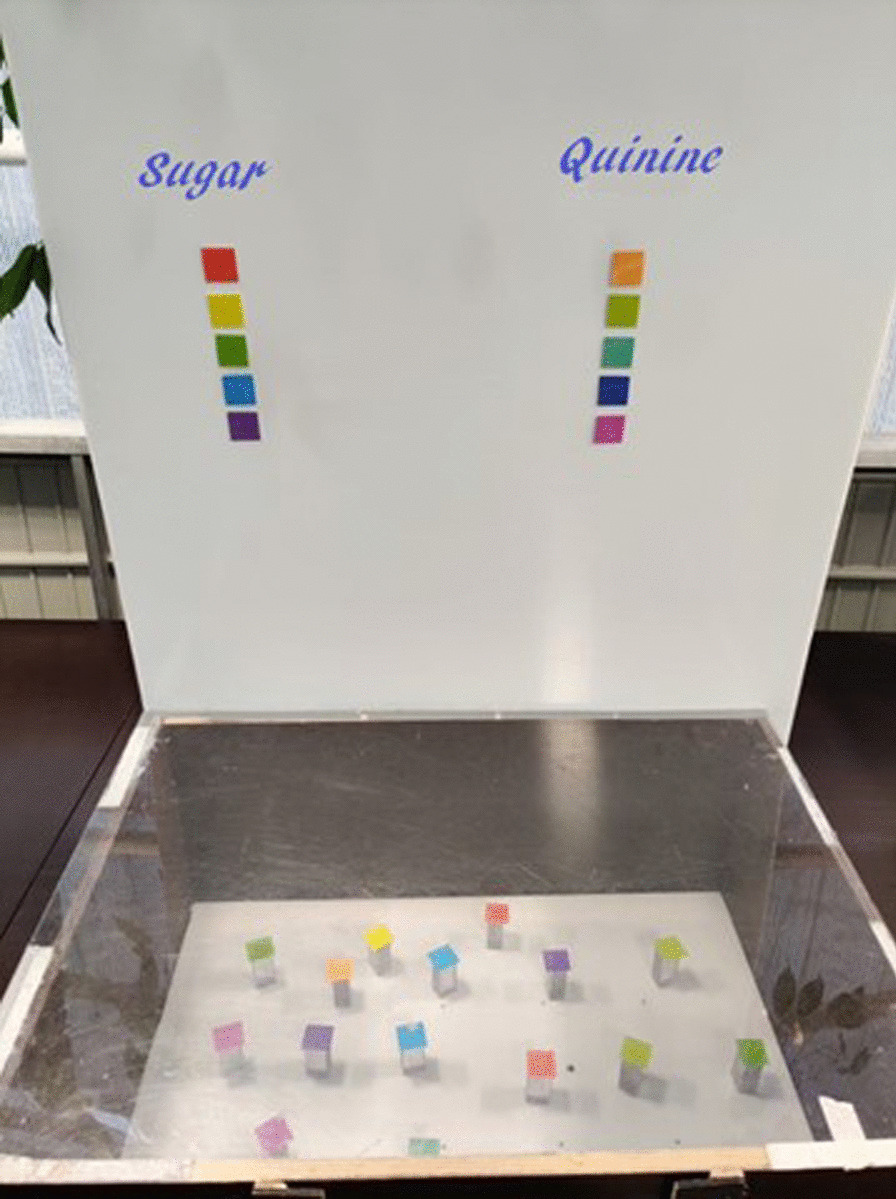
Flight arena with the colour chips. The colour patches under the titles ‘sugar’ and ‘quinine’ indicate whether the chips of the respective colours had 7 µl 40% sucrose solution or saturated quinine solution

In each learning bout, 20 coloured chips, two of each colour, were randomly placed in the flight arena; ten chips containing 7 µl 40% sucrose solution and ten chips containing 7 µl quinine (saturated in water) (Fig. 3). The bees were trained to discriminate colours by rewarding them (sucrose solution) or by punishing them (aversive quinine). The bees executed one bout until all rewarding chips were foraged, or ten minutes timeline was reached. A landing was defined as anytime a bee was positioned on the top of a chip and stopped flying and touched the sucrose/quinine with its antennae or proboscis. The landings were considered the decisions of a bee, and the proportion of landings to the rewarding colour chips was used as a response variable in models 1 and 2. The chips were equal in size (2.5 cm × 2.5 cm, height 5 cm), and the chips and the arena were cleaned with 70% ethanol after each bout.

The learning phase consisted of five bouts, the intervals between the learning bouts being a minimum of 10 min. Contrary to Li et al. [26], the interbout intervals were not consistent, because the foraging motivation of the bees varied.

#### Memory test

After the fifth learning bout, the bees were confined to the nest for two days, though having ad libitum access to the sucrose solution gravity feeder in the hall or corridor. The memory test was done after 2 days of confinement. The test was identical to one learning bout, though only water was used in all chips instead of sucrose and quinine liquids.

### Observation of symptoms after the higher pesticide exposure

The bees of the treatment 2 were observed for 1 h after the thiacloprid exposure. The symptoms were documented 5 min after the exposure and then in 10 min intervals. The symptoms were classified into five categories (1) ‘on its back’ when the bee laid either immobile or twitched on its back, (2) ‘actively flies or walks’ when the bee was actively flying or walking in the arena, (3) ‘vomiting’ when the bee was vomiting during the 10 min observation period, (4) ‘uncontrolled proboscis extension’ when the proboscis of the bee was hanging during the 10 min observation period, and (5) ‘passive’ when the bee did not move but stayed in place the whole 10 min observation period (Fig. 2).

### Statistical analysis

Statistical analyses were conducted to compare the performance of bees of treatments 0 and 1 in the learning phase and their memory retention from the fifth learning bout to the memory test. The analyses were conducted with R version 4.1.1 [33]. Generalized linear mixed-effects models and generalized linear models (GLMM ‘glmmTMB’functions in package lme4 [4]) were used. Two models were derived. The relative influence of each observation was adjusted in the models by using the ‘weights’ function.

Model 1. (GLMM) with the binominal distribution was used to test whether the treatment (control vs treatment 1; fixed factor), and its interaction with the bout number (learning bouts 1–5) (fixed factor) affected the performance of the bees (proportion of correct landings; response variable) in the learning phase. Colony and bee identity were used as random factors.

Model 2. (GLMM) with the binominal distribution was used to test whether the treatment (control vs treatment 1; fixed factor) and its interaction with the bout number (learning bout 5 and the memory test) (fixed factor) affected the memory retention of the bees (proportion of correct landings; response variable) from the fifth learning bout to the memory test. Colony and bee identity were used as random factors.

## Data Availability

The datasets analysed during the current study are available from the corresponding author on reasonable request.
